# Postural Stability after Unicondylar Knee Arthroplasty and Patient-Specific Interpositional Knee Spacer

**DOI:** 10.1155/2017/5836025

**Published:** 2017-07-13

**Authors:** J. Goetz, M. Baeurle, S. Dullien, J. Grifka, F. Koeck, C. Baier

**Affiliations:** Department of Orthopaedic Surgery, Regensburg University Hospital, Regensburg, Germany

## Abstract

*Purpose and Hypothesis. *Knee osteoarthritis results, inter alia, in decreased postural stability. After arthroplasty, postural stability recovers, but it is unclear whether this can be ascribed to a reduction of pain or to the preserving of receptor-rich intraarticular soft tissue and natural knee kinematics. The objective of this study was to evaluate whether an unicondylar knee arthroplasty provides better results regarding postural stability or a patient-specific knee spacer.* Methods*. In this comparative study, we assessed functional results and postural stability 16 months after 20 unicondylar knee arthroplasties (group A) and 20 patient-specific interpositional knee device implantations (group B). Patients were evaluated using the KSS and WOMAC score. Postural stability was analysed during single leg stance on a force platform (Biodex Balance System).* Results. *Concerning postural stability, range of motion (ROM), and KSS 16 months after the procedure, there were no significant differences between both groups.* Conclusion*. Successful treatment of knee osteoarthritis restores postural stability to the level of the contralateral side, regardless of the implant device.

## 1. Introduction

Knee osteoarthritis is the result of degeneration of articular cartilage and can affect any or all of the compartments of the knee. One-third of all concerned patients suffer from osteoarthritis solely of the medial compartment [1, 2]. Unicondylar knee arthroplasty (UKA) is an attractive option in progressive intraarticular unicompartmental osteoarthritis. Concerning functional scores and patients' satisfaction, it often leads to superior results compared to total knee arthroplasty despite a higher revision rate [3, 4]. Nevertheless, survivorship rates of 94% to 97% after 10 years have been reported [5]. Preserving both cruciate ligaments and bone is crucial factor for the reconstruction of knee kinematics. Studies revealed an almost physiologic movement pattern, with a primarily rollback mechanism of the lateral femoral condyle combined with tibial internal rotation during weight-bearing flexion after unicondylar knee arthroplasty [6]. Literature describes a more natural-feeling knee because of the preservation of proprioceptive tissue [7].

**Figure 1:**
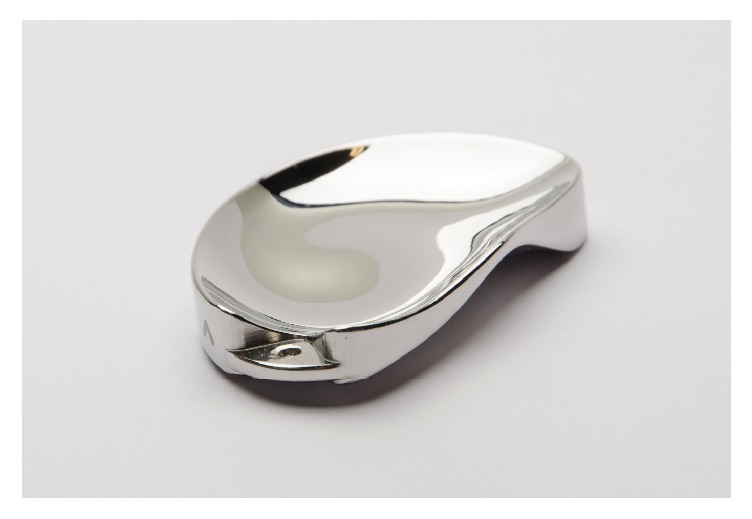
Interpositional knee device (ConforMIS iForma).

Knee spacers, introduced by Macintosh and later by McKeever [8, 9], have gained popularity since the 1950s. Different types of interpositional implants have been developed. While unicompartmental arthroplasty as a less invasive treatment option (compared to total knee arthroplasty) still requires bone resection, interpositional implants aim for preserving bone and delaying the need for a knee replacement [10]. Different types (ConforMIS iForma™, UniSpacer, and Orthoglide) which are varying in fixation and production (prefabricated versus custom manufactured) have been available (Figure 1). Although some authors [11, 12] revealed good results, it must be stated that these are also associated with high revision rates and poor outcome [13].

Published data concerning proprioception and postural stability show no significant differences between patients with a unicompartmental or a total knee implant [14]. In a second study, we were able to show that there were no significant differences between implantation of a knee spacer and total knee replacement [15].

No data exist about proprioception or postural stability after implantation of a (patient-specific) knee spacer in comparison with postural stability after unicondylar knee arthroplasty.

## 2. Purpose and Hypothesis

The purpose of this study was to compare postural stability together with functional results after unicompartmental knee arthroplasty and patient-specific interpositional knee spacer.

We hypothesized that a superior outcome after patient-specific knee spacer implantation is due to better proprioception and postural stability by preserving the natural anatomy.

## 3. Patients and Methods

### 3.1. Patients

This prospective study was designed to compare postural stability as well as functional knee status 16 months after either unicompartmental knee arthroplasty (UKA, Group A) or patient-specific interpositional knee spacer (iPD, Group B).

For iPD, the following inclusion criteria must be met: isolated unicompartmental knee osteoarthritis; fixed flexion deformity of less than 5°; an active range of motion (ROM) of greater than 90°; and less than 15° of varus deformity.

Inclusion criteria for UKA were the same; this procedure was performed in those patients who were not willing to receive an iPD. Exclusion criteria were metabolic diseases, endoprosthetic replacement of another joint of the lower extremity (both ipsi- or contralateral), rheumatoid arthritis, neurological diseases or deficiencies, vestibular deficiencies, preceding osteotomy, and revision surgery with exchange of components and any other type of orthopaedic surgery of the spine or lower extremities. Patients not willing to participate in the study were excluded as well. 20 patients met the inclusion criteria for UKA.

The number of the patients in reference group B (iPD) was adapted to the number of patients in group A (UKA). In total, 40 patients (*n* = 20 per group) were included in this study. The study protocol was approved by the local ethics committee (approval number 10-101-0240) and written informed consent was obtained from all participants.

### 3.2. Surgical Technique

The UKA were implanted using a standard medial parapatellar approach by three experienced surgeons (>100 UKA). One surgeon (F.K.) implanted all interpositional devices.

**Figure 2:**
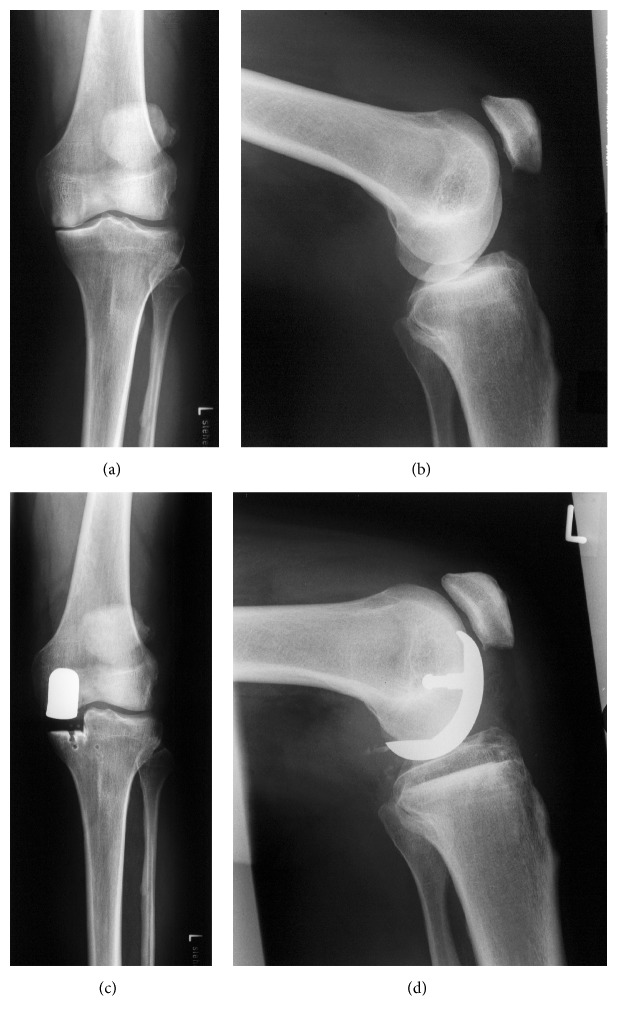
Unicompartmental knee (Preservation, DePuy, Warsaw, IN, USA), pre- and postoperative AP and lateral radiographs.

All patients from group A received a medial unicompartmental knee replacement, cemented with fixed platform (Preservation®, DePuy, Warsaw, IN, USA) (Figure 2).

**Figure 3:**
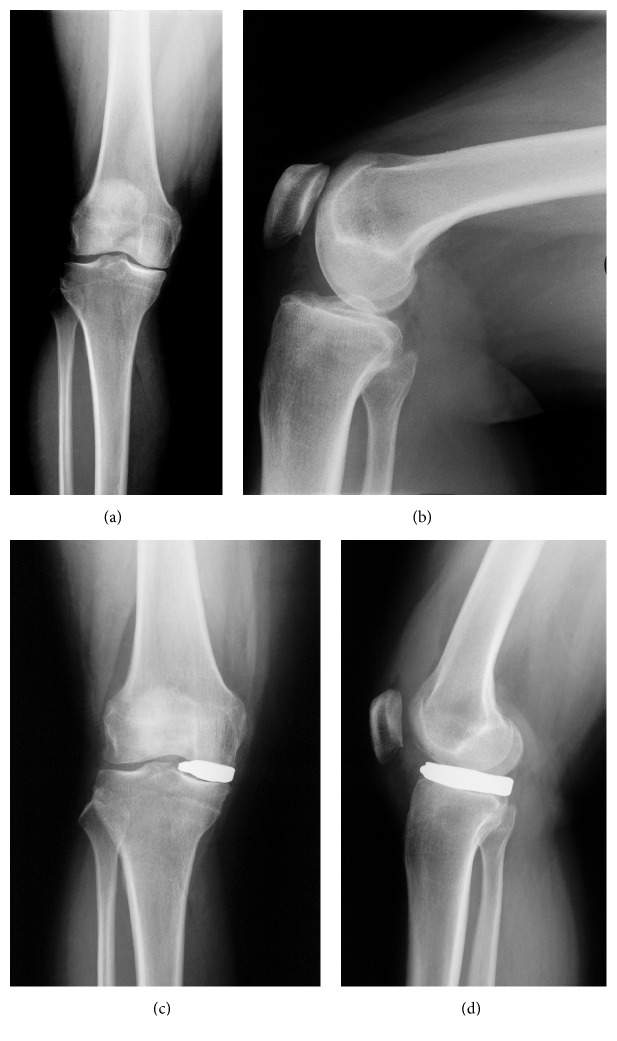
Patient-specific interpositional knee device (ConforMIS iForma), pre- and postoperative AP and lateral radiographs.

All patients from group B received a patient-specific interpositional knee device (ConforMIS iForma, Burlington, MA, USA) for the medial compartment (Figure 3).

The individualized knee spacer iForma was developed from a standard MRI scan using an image-to-implant technology that converts the topography of the patient's articular cartilage (thickness and curvature) and subchondral bone to a patient-specific implant considering the dimension of cartilage loss. In a one-stage procedure, the posterior horn of the medial meniscus was resected arthroscopically. A medial parapatellar miniarthrotomy was used to expose the compartment. Peripheral femoral and tibial osteophytes were resected as well as the rest of the medial meniscus; then the device was inserted using valgus stress and a special grasper. Intraoperatively implant stability was verified visually, by palpation and by dynamic fluoroscopy (Figure 3). Standard rehabilitation programs for any primary total knee arthroplasty were used for both groups, including full weight bearing limited by pain and no restriction of ROM.

### 3.3. Methods

All patients were evaluated 16 months after surgery. Patients' characteristics at follow-up evaluation included age, sex, and body mass index (BMI). The formula for determining BMI is weight (kg) divided by height squared (m^2^) (kg/m^2^). Moreover, knee status was assessed by Knee Society Score (KSS), Western Ontario McMaster Universities Osteoarthritis Index (WOMAC LK 3.1), and postural stability.

Biodex Balance System (Biodex Inc., Shirley, NY, USA) was used to measure postural stability. This device consists of a multiaxial unstable, but gradually lockable platform (diameter 55 cm), which is capable of tilting in the sagittal and transverse plane and a screen, located in head height. The platform measures and records the location of the center of balance (COB) of the person standing upright on the platform and displays it simultaneously on the screen. The maximum tilt of the platform is 20° from the horizontal position to all sides. The apparatus prompts participants to center a cursor, viewed on a liquid crystal display, representing the center of balance while standing on the measuring platform. By altering the resistance of the platform to deviations, the level of difficulty for the patients can be modified.

The ability to balance is expressed by a balance index as a mean deviation in degrees of three required trials, calculated by using the time and deviation. For data analysis, the medial/lateral stability index (MLSI), anterior/posterior stability index (APSI), and overall stability index (OSI) were recorded. The anterior-posterior index represents the distance of movement of the calculated COB along the sagittal plane, the medial-lateral index represents the distance of COB movement along the frontal plane position, and the OSI represents the variance in COB displacement across all directions of platform motion [16]. As OSI represents the global status of postural stability concerning all directions, we used it as key parameter for our assessment. The Biodex stability system has an interclass correlation coefficient ranging from *r* = 0.6 to *r* = 0.96 [17].

The first test was carried out on the unstable platform with both legs to rule out any balance disorders. Three trials were performed with a measurement time of 20 seconds of each trial. The patients had to keep their center of mass in the center of the target. The patient was positioned legs hips-width apart, knees slightly bended, and arms and hands held over the handlebars for security reasons. Patients were told not to use the handlebars during data recording. Afterwards the ability to balance on one leg was tested. The patients had to balance on one leg on the locked platform for 3 × 10 seconds. We started with the nonoperated leg. The standing leg was centered on the now locked platform and neither the other leg nor the hands were allowed to have contact with the system. The trial duration was set to 10 seconds and resting between trials was set to 15 seconds to prevent muscle fatigue. The test protocol as well as the examined parameters had been evaluated in other studies before [17, 18]. The overall stability index (OSI) was chosen as an important parameter, being a composite of MLSI and APSI and representing the global variance of platform displacements in all motions during a test.

## 4. Statistics

Statistical analyses were performed using SigmaPlot for Windows 12.0 (Systat Software, Chicago, IL). The significance level was set at *p* ≤ 0.05. Patients' characteristics were evaluated using descriptive statistics (frequencies [*n*], percentages [%], means [*m*], standard deviations [sd/±], medians [med], and percentiles [*Q*1, *Q*3]). Comparing both groups, *t*-test for normally distributed variables and Mann–Whitney *U* test for not normally distributed variables were used. Shapiro-Wilk test was used to assess the distribution of the metric variables. To assess associations between BMI with postural stability, Spearman's rank correlation coefficient was used.

## 5. Results

### 5.1. Sample Description

In total, 40 patients (55% female, *n* = 22) with knee osteoarthritis were included in this study. The mean age was 57.6 years (±7.99) and the median BMI 28.95 (*Q*1 = 27.0, *Q*3 = 30.9). Patients with unicondylar knee arthroplasty (UKA; group A) and patients with specific interpositional spacer (IKS; group B) did not differ in sex and BMI. There was no significant difference in preoperative ROM and axis deviation, either. However, both groups did significantly differ in age (*p* < 0.001). Group B was significantly younger [med (*Q*1, *Q*3) = 54 (47.25, 60.75)] than group A [med (*Q*1, *Q*3) = 62 (56.75, 65.75)]. Descriptive analyses are presented in Table 1.

### 5.2. Group Comparisons regarding Knee Status and Postural Stability

Concerning the knee score (UKA 85.75 ± 12.62; iPD 81.55 ± 12.33) and function score (UKA 81 ± 18.03; iPD 76.50 ± 15.31) of the KSS, no significant differences could be detected between both groups (*p* = 0.324, knee score; *p* = 0.276, function score). There was no statistic significant difference in WOMAC total scale. Regarding the subscales, the only statistical significant difference detected was in WOMAC pain.

The median overall postural stability index for the two-leg stance on the unstable platform, describing the variance of change of the platform in degree, was 1.2 in group A and 1.55 in group B. No significant differences were detected considering the mean postural stability between both groups (*p* = 0.232).

The differences in one-leg stance on the locked platform between operated side (OSOS) and nonoperated side (OSNS) were not significant. In group A, the value of the median was 1.4 at the operated side and 1.3 at the contralateral side (*p* = 0.871). In group B, the value of the median was 1.0 at the operated side and 0.95 at the nonoperated side (*p* = 0.935). The difference in one-leg stance at the operated side was not statistically significant comparing the medians between UKA (1.4) and iPD (1.0) (*p* = 0.193).

Regarding ROM after surgery, there were no significant differences between knee flexion of both groups (UKA 126.5  ±  8.75°, iPD 126.25  ±  9.85°, *p* = 0.948). In each group, three patients had a deficit in extension of 5° and one patient an extension deficit of 10°. No significant differences between both groups were detected concerning the circumference of thigh and shank at determined levels (thigh prox. *p* = 0.360, thigh dist. 0.931, shank *p* = 0.235).  Table 2 shows the results of knee status and postural stability.

Checking the influence of body mass index on postural stability, it could be proven that, in group A, a moderate correlation (Spearman's rank correlation: *r* = 0.323) existed concerning the interdependence between postural stability and body mass index. Also, in group B, we could detect a correlation between BMI and postural stability (*r* = 0.605) in two-leg stance. This means that an increase in BMI leads to worse results in postural stability in both groups, but statistically significant solely in the iPD group (*p* = 0.002).

### 5.3. Complications

No complications occurred in group A.

In group B, two of twenty patients had to undergo revision surgery after dislocation of the spacer, one because of an adequate trauma and the other because of dislocation due to impinging osteophytes. In both cases, the spacer could remain in place and the adverse events were not classified as exclusion criteria.

## 6. Discussion

It must be highlighted in advance that the purpose of this study was not to evaluate the status and importance of interpositional knee spacers, which show discouraging results in mid- to long-term follow-up, but to prove the influence of arthroplastic devices on postural stability. The currently increasing number of younger patients (i.e., <60 yrs) with unicompartmental osteoarthritis of the knee and a high activity level means a growing demand for surgical solutions, which respect the requirements of the patients. To fill the gap between biological cartilage repair and traditional uni- or bicondylar knee arthroplasty, femoral and high tibial osteotomies, as well as interpositional knee spacers, had gained popularity.

Excellent long-term results and patient satisfaction after UKA are ascribed to the preservation of both cruciate ligaments and bone stock. Despite superior clinical and functional results over TKA [4], unicompartmental knee resurfacing does not comprise better proprioception [19, 20] than TKA.

To our best knowledge, there do not exist any data on postural stability of patients after implantation of a (patient-specific) interpositional knee device compared with unicompartmental arthroplasty.

There was a statistical significant difference in both groups concerning age. This was due to the fact that the interpositional knee spacer was used particularly in younger patients. In order to provide comparability, we evaluated the condition of the patients using clinical scores (WOMAC, KSS) and measuring the range of motion. We detected that there were no significant different results regarding KSS and WOMAC total scale. The only significant difference was found in the WOMAC subscale pain with significant better results of the UKA. The range of motion did not show any significant difference.

The primary task of the above-mentioned clinical outcome was to ensure data comparability of both cohorts. However the focus of our study was to examine the influence of different surgical solutions for knee osteoarthritis on postural stability. By preserving the natural anatomy of the patient's knee with the patient-specific interpositional implant, we hypothesized that postural stability will be superior to UKA. Our results, however, showed no significant differences between both groups.

Proprioceptive abilities following total knee arthroplasty have mostly shown inferior results compared to healthy age-matched control persons [21]. However, compared to osteoarthritic age-matched control persons, they achieve better capabilities [22, 23]. Literature reveals that loss of proprioception is independent of the severity of knee osteoarthritis and, respectively, unilateral knee osteoarthritis impairs proprioceptive accuracy in both knees [23, 24].

Regarding these facts, you can assume two effects concerning postural stability and proprioception after patient-specific knee interpositional device implantation: first, it may provide inferior results due to a probable neglectable influence on leg axis, not influencing the process of knee osteoarthritis at all and being estimated as a foreign body by the patient. Second, it may provide better results due to preserving the natural anatomy, trying to fill out cartilage defects by its shape, the resection of osteophytes, and the proven possibility of sufficient leg axis correction [25]. In our cohort of individualized knee spacers, we could detect a distinct better postural stability, however not being significantly better than after UKA.

Measurement of postural stability after implantation of either individual knee spacer or unicompartmental knee replacement as an instrument of postoperative follow-up examination by using only double-leg stance may be discussed controversially obtaining a result that is influenced by the operated knee and by the contralateral side. But it is a good instrument as entry examination to rule out balance disorders or any other impairment which could bias the results.

By using single leg stance as a test for evaluation of postural stability, the influence of the nonoperated side can be eliminated. Therefore, we chose this kind of test as it is more meaningful to compare the two different implants. Nevertheless, the deficits of the setup have to be considered, for example, the task of maintaining in-place balance while standing is different from maintaining balance while walking. On closer examination of the results of both groups and neglecting individual results, the statistical spread in both groups (UKA, iPD) is in accordance with the statistical spread of healthy subjects [26]. Therefore, the achievements of the sensomotoric system of the patients feature the identical variations, which may occur in interindividual comparison of healthy subjects. In total, the system used in our study represents an established and commonly used procedure.

Apart from design, an adverse effect of postural stability by the implant type could not be proven. Our study shows that the use of an interpositional knee spacer as well as the implantation of an unicompartmental replacement restored postural stability in one-leg stance so that no significant differences could be detected between both groups regarding the operated side. To our best knowledge, similar studies do not exist in literature so far. Regarding the results of postural stability after total knee replacement and the influence of substituting or retaining the posterior cruciate ligament, it could be shown that, similar to the current study, patient regained the status of the nonoperated side independently from the surgical method [20]. Furthermore we could show that, in both groups, the overall postural stability of the operated side reached the level of the nonoperated side. Several factors and structures around the knee contribute to proprioception and postural stability after TKA like collateral ligaments, periarticular muscle tendons, and the joint capsule [19]. Pain, inflammation, deviation of axis, joint space narrowing, and nonphysiologic kinematics affect postural stability additionally in the arthritic joint. Sánchez-Herán et al. reports that pain catastrophizing and fear-avoidance beliefs are related to postural stability, too [27]. Pain relief, restoring the physiological periarticular soft tissue tension and reconstruction of leg axis seems to influence postural stability more than the choice of the implant or the preservation of receptor-rich intraarticular tissue. The role of the periarticular tissue must be highlighted even in the context of obesity. We could show once more that the increase of BMI inversely correlates with postural stability [20]. However, the relevance of this result remains undetermined. Because of methodical limitation, the interpretation remains unclear if this result is directly caused by obesity or indirectly because obesity per se makes it difficult to balance.

The measurement of circumference of thigh and shank showed no statistically significant differences between both groups. The interpretation of the measurement of circumferences, however, should be done with caution. The circumference of thigh and shank does neither reflect atrophy of muscle nor strength. The interpretation of outcome of knee arthroplasty based on the measurement of leg circumference as a single instrument is not sufficient and the interpretation must be done in conjunction with other functional results.

Regarding the results of our study, the two types of implants (UKA, iPD) are equivalent in relation to postural stability and range of motion, as well as circumference of shank and thigh and WOMAC and KSS score. The UKA group showed better results in the subscale WOMAC pain than the iPD group.

Although inclusion and exclusion criteria were composed very strictly and there does not exist any literature about postural stability after implantation of an interpositional knee device, this study has its limitations, as it is not randomized and no preoperative status regarding postural stability was detected. The follow-up period was 15.7 months. The most improvement in proprioception and gait occurs within the first 6 months after knee replacement surgery [28, 29]; after that period, it could be possible that the effect of proprioception is compensated by other neuromuscular mechanisms. There is a difference in age in both groups originating from the fact that the indication for an interpositional knee spacer was seen in younger patients, preserving the complete bone stock and having all treatment options later on. This fact must be considered responsible for the significant differences on overall postural stability between the two groups.

Furthermore, the patient-specific interpositional knee device does not completely preserve the natural anatomy. It rather reflects “osteoarthritic anatomy” being based on MRI scans of osteoarthritic knees. However, this individualized implant tries to fill cartilage bone defects by its shape. Furthermore, peripheral osteophytes were removed during implantation. No kinematic results based on fluoroscopy or in vivo navigation exist so far to confirm in vitro results of preserving knee kinematics with interpositional knee spacers. Whenever performing the implantation of a unicompartmental or even a total knee replacement, you have to consider the inevitable loss of bone stock. Although being an appropriate option for delaying the implantation of a knee replacement, the distribution of the spacer used in this study has been discontinued by ConforMIS.

## 7. Conclusion

We could not detect better postoperatively results concerning postural stability comparing interpositional knee spacers with UKA. If anything, patients showed significantly better results concerning WOMAC subscale pain after UKA. Successful treatment of knee osteoarthritis restores postural stability to the level of the contralateral side, regardless of the implant device used in this study.

## Figures and Tables

**Table 1:** Descriptive analyses.

*N* = 40	Group A UKA^1^ (*n* = 20)	Group B iPD^2^ (*n* = 20)
Sex		
Male (*n* [%])	9 (45%)	9 (45%)
Female (*n* [%])	11 (55%)	11 (55%)
Age (*m* ± sd)	61.72 ± 4.83	53.85 ± 8.63
BMI (med [*Q*1, *Q*3])	29.3 (26.9, 30.7)	28.70 (26.50, 32.68)

^1^UKA = patients with unicompartmental total knee arthroplasty, ^2^iPD = patients with specific interpositional spacer.

**Table 2:** Group comparisons regarding knee status and postural stability.

*N* = 40	Group A UKA^1^ (*n* = 20)		Group B iPD^2^ (*n* = 20)		*p* Value
*Knee Society Score (KSS)*	*m* ± sd		*m* ± sd		*t*-tests

Knee score	85.75 (12.62)		84.20 (11.75)		0.324
Function score	81.5 (18.43)		76.50 (15.31)		0.276

*Western Ontario McMaster Universities Osteoarthritis Index (WOMAC)*	med (*Q*1, *Q*3) sum of score	*m* ± sd mean of items	med (*Q*1, *Q*3) sum of score	*m* ± sd mean of items	*U*-tests (sum of score)

Total	13.0 (6.00, 34.50)	0.83 ± 0.63	27.0 (9.25, 47.00)	1.17 ± 0.78	*p* = 0.144
Pain	3.0 (0.25, 4.0)	0.57 ± 0.5	5.0 (2.0, 7.75)	1.1 ± 0.8	*p* = 0.033
Stiffness	2.0 (0.25, 3,75)	1.13 ± 0.9	3.0 (0.25, 4.0)	1.23 ± 0.9	*p* = 0.680
Function	9.5 (4.0, 24.25)	0.81 ± 0.7	18.0 (6.25, 35.0)	1.18 ± 0.9	*p* = 0.147

*Range of motion (ROM)*	*m* ± sd		*m* ± sd		*t*-tests

ROM knee (extension/flexion)	126.5 (8.75)		126.25 (9.85)		0.948

*Overall postural stability index (OSI)*	med (*Q*1, *Q*3)		med (*Q*1, *Q*3)		*U*-tests

*Two-leg stance*	1.2 (1.00, 2.10)		1.55 (1.13, 2.23)		0.232
*One-leg stance on operated side (OSOS)*	1.4 (0.85, 1.80)		1.00 (0.80, 1.45)		0.193
*One-leg stance on nonoperated side (OSNS)*	1.3 (1.05, 1.55)		0.95 (0.83, 1.10)		0.042
*U-test (OSOS : NSOS)*	0.871		0.935		

^1^UKA = patients with unicompartmental knee arthroplasty. ^2^iPD = patients with specific interpositional spacer.

## Abbreviations

APSI: Anterior/posterior stability index
COB: Center of balance
iPD: Interpositional device
MLSI: Medial/lateral stability index
OSI: Overall stability index
ROM: Range of motion
TKA: Total knee arthroplasty
UKA: Unicompartmental knee arthroplasty.
